# Comparison of Post-Consumption Attention Between Two Different Aroma Types of Baijiu Using the Stroop Task

**DOI:** 10.3390/foods14132352

**Published:** 2025-07-02

**Authors:** Jie Li, Cheng Wang, Pei Sun, Shaohui Fan, Guoliang He, Yangyun Zheng, Haifeng Zhao, Deliang Wang

**Affiliations:** 1 School of Food Science and Engineering, South China University of Technology, Guangzhou 510640, China; 2 China National Research Institute of Food and Fermentation Industries Co., Ltd., Beijing 100015, China; 3 Guangdong Shiwan Baijiu Group Co., Ltd., Foshan 528031, China; 4 Shiwan Qingya Baijiu Research Institute, Foshan 528031, China; 5 Faculty of Health and Wellness, City University of Macau, Macau SAR 999078, China; 6 Department of Psychology, Tsinghua University, Beijing 100084, China

**Keywords:** Chinese baijiu, attention, Stroop, EEG, post-drinking comfort

## Abstract

As attention reflects cognitive comfort after drinking, and consumers increasingly value mental clarity, there is a growing need for objective methods to quantify such post-consumption cognitive states. However, conventional expert sensory panels rely on subjective judgments and cannot objectively quantify post-consumption changes in attention. To address this gap, this experimental study applied and validated an established electroencephalogram (EEG)-based behavioral index and then investigated whether two aroma types of Chinese Baijiu with equal alcohol content differ in their effects on attention. Twenty-one adults completed the color–word Stroop task, and behavioral performance and neural responses were recorded before and after drinking. The results showed that despite the same alcohol content, different aroma types of Baijiu had varying effects on the accuracy of incongruent conditions in the behavioral task. Additionally, they exerted varying effects on the magnitude of the P200 component, an attention-related EEG signal typically occurring around 200 ms after stimulus onset. The light-aroma Baijiu (Sample 2) significantly reduced task accuracy and P200 amplitude, whereas the Qingya-flavored Baijiu (Sample 1) had no significant impact. These findings provide preliminary evidence that attention-based EEG behavioral metrics can serve as an objective reference for evaluating post-consumption cognitive quality and may inform product optimization and consumer-centered quality standards in the Baijiu industry.

## 1. Introduction

Chinese baijiu is a high-proof spirit made from grain through solid-state fermentation and distillation processes. Compared with wine and beer, it has a higher ethanol concentration (typically~42–60% ABV) and contains a broader range of fermentation-derived compounds such as esters, higher alcohols, and aldehydes, many of which are known to influence not only flavor but also metabolic and physiological responses [1]. Different aroma types of Chinese Baijiu can lead to distinct sensory perceptions and consumer experiences, as they differ significantly in their chemical composition [2]. With the rise of health-conscious consumption, the focus of alcoholic beverage evaluation has gradually shifted from flavor and taste to broader post-drinking experiences [3]. Among these, post-drinking comfort has emerged as a widely recognized concern among Chinese consumers and is often regarded as a practical criterion for evaluating product quality and shaping purchasing decisions. Unlike the commonly studied concept of hangover, which reflects a set of overt negative effects following heavy alcohol intake [4], post-drinking comfort refers to the overall physiological and psychological state following moderate alcohol consumption, encompassing both positive and negative influences on physical, emotional, and cognitive functions such as attention and memory [5,6,7]. However, despite its growing importance in consumer experience, post-drinking comfort remains underexplored in empirical research, especially in the context of Chinese Baijiu and its varied aroma profiles.

Existing studies on Chinese Baijiu have mainly focused on sensory flavor analysis [8,9,10,11], relying largely on expert-based subjective evaluation methods, while lacking objective and physiological validation, particularly concerning the cognitive aspects of post-drinking comfort [12]. Although experts have advantages in providing detailed and in-depth evaluations, there are still several limitations. First, due to differences in individual taste perception and preference, the opinions of experts can not represent the real attitude of ordinary consumers [13]. Second, sensory evaluation methods are highly subjective, and even when evaluating the same content, different individuals will have different understandings of its definition due to cultural background, life experience, and value judgments, making it difficult to unify sensory thresholds and evaluation standards [14,15]. Third, expert sensory evaluation panels usually consist of a small number of people, which poses significant difficulties for statistical analysis and limits the generalizability and applicability of conclusions [16]. Therefore, an urgent need exists for a more subtle evaluation framework grounded in objective physiological and cognitive indicators to assess post-drinking comfort from the consumer’s perspective in Chinese Baijiu.

Attention is a key cognitive aspect of post-drinking comfort in the context of alcohol consumption. It is a complex cognitive process in the brain, aimed at optimizing resource allocation [17,18] and improving information processing efficiency, enabling humans to better adapt to the living environment. In neuroscience, selective attention is defined as the brain’s ability to selectively process external stimuli while ignoring other irrelevant interference information [19]. Alcohol can affect attention to a certain extent, and this effect is usually negative [20]. After alcohol consumption, the inhibitory neurotransmitter (GABA) increases, while the excitatory neurotransmitter (glutamate) decreases [21] and this change inhibits the activity of the relevant brain regions responsible for the allocation and regulation of attention resources (such as the frontal lobe), thereby reducing an individual’s ability to concentrate, manifested in behaviors such as slower information processing speed and decreased reaction accuracy. Under the premise of moderate drinking, high-quality alcoholic beverages should not significantly impair such an important cognitive function as attention. Therefore, evaluating changes in consumers’ attention before and after drinking can objectively reflect the cognitive quality of post-drinking comfort for alcoholic beverages.

The Stroop color–word task is a classic paradigm in psychology for evaluating selective attention and cognitive control [22,23,24]. Psychologist John Ridley Stroop found that most people respond significantly slower when the meaning of a word does not match the color of the printed word (e.g., the word “blue” printed in red), which is known as the Stroop effect [25]. Subsequent studies expanded on this research, using response accuracy as an important indicator [17,23,26,27]. In addition, the Stroop task has been widely used in the evaluation and diagnosis of clinical psychiatric and neurological disorders, such as attention deficit hyperactivity disorder (ADHD), Alzheimer’s disease, and post-traumatic stress disorder (PTSD) [28,29,30].

Recently, EEG technology has garnered increasing attention in consumer science research and has shown great application potential [31]. EEG can non-invasively measure local field potential generated by synchronized neuronal activity in the brain at a temporal resolution of milliseconds, making it suitable for studying various cognitive functions such as attention, memory, and emotion [32]. Alvino et al. used EEG to investigate consumers’ behavior and preference during wine consumption and found that the higher the preference for wine, the greater the decrease in β oscillation [33,34,35]. EEG power was used to predict consumers’ decisions when viewing mobile product advertisements, with an accuracy exceeding 87% [36]. Moore et al. [37] used the Stroop task as a measure of attention control, finding that EEG features in the 160–240 ms after stimulus presentation reflected improved attention following meditation training.

Nevertheless, little is known about how different aroma types of Chinese Baijiu acutely influence EEG measures of selective attention collected shortly after drinking, leaving an important gap in our understanding of the cognitive brain mechanisms that underpin post-drinking comfort. To address this gap, the present study combines the Stroop color–word task with continuous EEG recording to objectively assess attentional changes before and after the consumption of different Baijiu. We hypothesize that Baijiu aroma types will differentially affect both behavioral performance and event-related potential (ERP) components associated with selective attention, particularly the amplitude of responses around 200 ms. These findings will advance research on the cognitive aspects of post-drinking comfort in alcoholic beverages, establish a scientific foundation for product quality evaluation, and promote health-conscious consumption.

## 2. Methods

### 2.1. Subjects

This study was approved by the ethics committee of Tsinghua University and carried out according to the Declaration of Helsinki. Twenty-one right-handed subjects (including 7 females, *M* = 24.9 years, *SD* = 4.3) participated in this experiment. The sample size is consistent with previous EEG Stroop studies [37,38]. An a priori power analysis using G*Power version 3.1 (α = 0.05, two-tailed, *d*_z_ = 0.65) indicated that the number of participants would provide 80% power to detect medium effects in paired-samples *t*-tests.

Screening criteria included not having neurological or psychiatric disorders and not being allergic to alcohol. All subjects had normal or corrected-to-normal vision and were not color-blind. They signed informed consent forms before the experiment and received monetary compensation afterward. All subjects were included in behavioral and physiological analyses, while two were excluded from the EEG analysis due to technical issues.

### 2.2. Materials

Subjects consumed two different aroma types of 53-degree Baijiu during the study. One was Qingya-flavored Baijiu (sample 1), and the other was a typical light-aroma Baijiu (sample 2). These two aroma types were selected because Qingya-flavored Baijiu is an emerging style closely related to the traditional light-aroma category, whereas light-aroma Baijiu is long-established and widely consumed, providing a meaningful contrast. Both samples were sourced from the same shopping website and were easily available. They were packaged in glass bottles and stored under the same conditions to ensure consistent testing conditions. Each subject consumed an appropriate amount of alcohol based on their body weight (0.7 g/kg) [39,40], with an intake ranging from 45 to 75 mL, following a standardized protocol and speed of consumption. To avoid brand effects on experimental results [41,42,43], both samples were provided in identical unmarked containers, and the subjects were not informed about the samples during the experiment.

### 2.3. Stimuli

**Figure 1:**
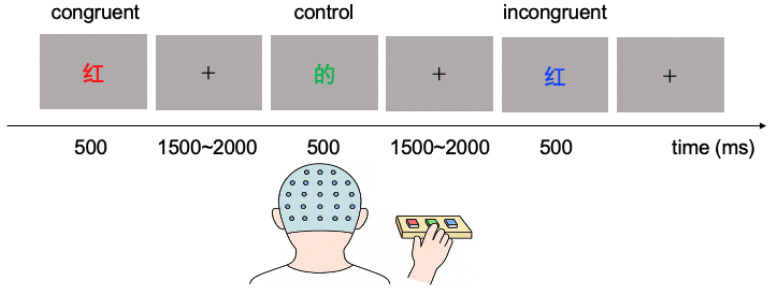
The Stroop color–word task. The subjects should press the keys that are consistent with the printed color of the word as quickly and accurately as possible.

The subjects performed a Stroop color–word task during the experiment (Figure 1). Simplified Chinese characters in three different colors—‘红’ (red), ‘绿’ (green), ‘蓝’ (blue)—were presented on a computer screen in a pseudo-random order. One type of words was color-related (e.g., ‘红’, ‘绿’, ‘蓝’), and the other type was neutral words (e.g., ‘的’: ‘of’, ‘则’: ‘thus’) that are semantically unrelated to color. These neutral words were chosen because they match the color words in overall structure and stroke count [44]. In the congruent condition, the printed color of the word matched its meaning (e.g., the word ‘蓝’ was printed in the color blue, and the word ‘红’ was printed in the color red). In the incongruent condition, the word’s printed color did not match its meaning (e.g., the word ‘红’ was printed in the blue color, and the word ‘蓝’ was printed in the green color). In the control condition, neutral words were shown in the three font colors so that the characters themselves conveyed no color information. Each stimulus was presented for 500 ms, with an inter-stimulus interval (ISI) of 1500–2000 ms. The program for presenting stimuli and recording responses was written with MATLAB (MathWorks, version 2019a) language and Psychtoolbox 3.0.16 [45].

### 2.4. Procedure

Each subject took part in two experimental sessions, spaced one week apart, consuming one sample of Baijiu in each session, with the order randomized. At the start of each session, behavioral and EEG data were collected as the subjects performed the Stroop color–word task before drinking. They were instructed to selectively attend to the color of the words while ignoring their meaning and to respond by pressing the corresponding color button as quickly and accurately as possible. The task included 180 trials across three experimental conditions. The subjects then consumed 1/3 of the total alcohol dose every 10 min under supervision and guidance, with skin temperature and heart rate recorded. They were required to complete the specified amount of alcohol intake within 30 min and then perform the Stroop task again after the drinking, following the same procedure as the pretest. Continuous EEG data were recorded throughout the process.

### 2.5. EEG Recording and Preprocessing

The EEG signals were collected using a 64-channel Neuroscan system with the SynAmps amplifier II. The Ag/AgCl cap electrodes were arranged according to the International 10–20 system, referenced online to CZ. Data were sampled at 500 Hz, with electrode impedance kept below 10 kΩ. Subjects were asked to keep their body and head still during the tasks, and swallow and blink during the stimulus interval to minimize artifacts. An independent component analysis (ICA) algorithm was used to remove artifacts such as eye movements and blinks [46]. The EEG were band-pass filtered 1 to 45 Hz offline and re-referenced to the average of all the electrodes. Epochs were segmented from 200 ms before to 1000 ms after stimulus onset, with a 200 ms pre-stimulus baseline correction. Based on prior evidence that the ERP peaks at the mid-frontal site FCZ in Stroop paradigms [38,47], data analyses were confined to this a priori region of interest. This choice also supports future application in low-channel wearable EEG systems.

### 2.6. Statistical Analysis

To assess the impact of different intake amounts of both samples on physiological indices (skin temperature and heart rate), a two-factor ANOVA of repeated measures was conducted. One factor was alcohol consumption, which had four levels: 0 before drinking, 1/3 of the total amount consumed within 10 min, 2/3 of the total amount consumed within 20 min, and the entire amount consumed within 30 min. The other factor was sample type, which had two levels: Sample 1 and Sample 2. Mauchly’s test was used to evaluate the assumption of sphericity, and if violated, the Greenhouse–Geisser correction was applied. Post hoc analysis was conducted using Tukey’s Honest Significant Difference (HSD) test.

Given that the primary interest of this study is to investigate whether alcohol significantly affects attention-related behavioral and neural responses under specific experimental conditions, the statistical methods employed for analyzing behavioral and EEG data were based on explicit pre-planned tests. Paired *t*-tests were used to analyze the differences in behavioral indices (accuracy and response time) and ERPs (about 200 ms) between pre-drinking and post-drinking for the same subjects under congruent, control, and incongruent conditions separately. To control Type I errors, the False Discovery Rate (FDR) method was used for multiple corrections. The significance level of the statistical tests was set at 0.05.

## 3. Results

### 3.1. Physiological Results

**Figure 2:**
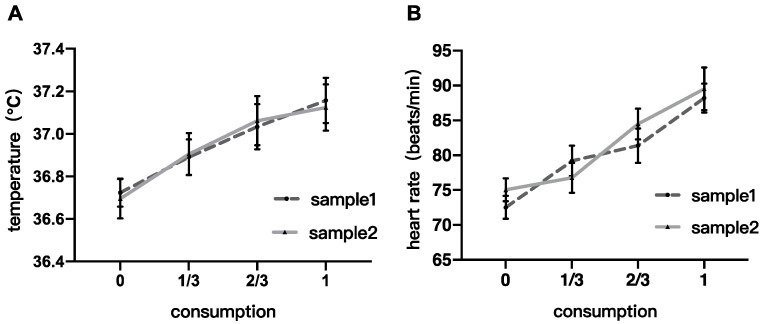
Physiological results. (**A**) skin temperature changes with alcohol consumption; (**B**) heart rate changes with alcohol consumption.

Figure 2 shows the physiological indicators (skin temperature and heart rate) of the subjects as they change with alcohol consumption. A two-factor repeated measures ANOVA was conducted to investigate the effects of alcohol intake and sample type on skin temperature and heart rate.

The analysis of skin temperature revealed a significant main effect of alcohol consumption, *F*_(3,60)_ = 20.105, *p* < 0.0001, *η*^2^ = 0.060. The main effect of sample type and the interaction between the two factors were not significant (*F*_(1,20)_ = 0.008, *p* = 0.932, *η*^2^ = 0.00003; *F*_(3,60)_ = 0.305, *p* = 0.822, *η*^2^ = 0.122, respectively). Tukey HSD post-hoc analysis showed significant changes in skin temperature at two-thirds consumption (*p* = 0.004) and after complete consumption (*p* = 0.001) compared to pre-drinking, with no significant differences at other stages (*p*s > 0.05).

The analysis of heart rate also showed a significant main effect of consumption, *F*_(3,60)_ = 17.097, *p* < 0.0001, *η*^2^ = 0.120. The main effect of sample type was not significant (*F*_(1,20)_ = 0.830, *p* = 0.373, *η*^2^ = 0.002), and the interaction between the two factors was also not significant (*F*_(3,60)_ = 0.972, *p* = 0.412, *η*^2^ = 0.251). Post-hoc analysis showed significant differences in heart rate between all stages (*p*s < 0.05) except between one-third consumption and pre-drinking, and between one-third and two-thirds consumption.

Physiological analysis results suggested that the main factor influencing skin temperature and heart rate during drinking is the amount of alcohol consumed, with similar patterns observed for both samples and no statistically significant differences between them.

### 3.2. Behavioral Results

The accuracy and response time results in the congruent, control, and incongruent conditions of the Stroop color–word task during the pre-drinking and post-drinking phases were summarized in Table 1 below.

**Figure 3:**
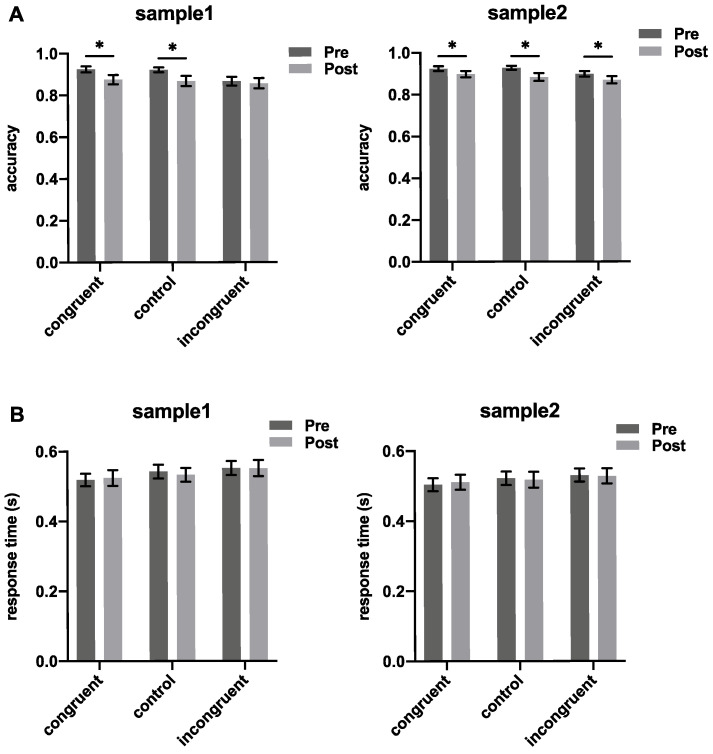
Accuracy and response time of both samples under the three experimental conditions during the pre-drinking and post-drinking phases. (**A**) accuracy results; (**B**) response time results. One-sided paired *t*-tests, multiple corrections using FDR method, significance level set at 0.05, * indicates *p* < 0.05.

Paired *t*-tests were conducted on the pre-test and post-test results of behavioral indicators, with multiple corrections performed using the FDR method. The results of the accuracy analysis are shown in Figure 3A. For Sample 1, the decrease was statistically significant in the congruent condition (*t*_(20)_ = 2.226, *p*_corrected_ = 0.028) and the control condition (*t*_(20)_ = 2.440, *p*_corrected_ = 0.028). However, the decrease was not significant in the incongruent condition (*t*_(20)_ = 0.546, *p*_corrected_ = 0.296). For Sample 2, the accuracy significantly decreased in all conditions during the post-drink phase (congruent: *t*_(20)_ = 1.737, *p*_corrected_ = 0.049; control: *t*_(20)_ = 2.794, *p*_corrected_ = 0.019; incongruent: *t*_(20)_ = 2.449, *p*_corrected_ = 0.018).

The response time analysis results are shown in Figure 3B. For Sample 1, response times increased in the post-drink phase across all conditions but were not significant (congruent: *t*_(20)_ = −0.509, *p*_corrected_ = 0.692; control: *t*_(20)_ = 0.910, *p*_corrected_ = 0.560; incongruent: *t*_(20)_ = 0.060, *p*_corrected_ = 0.6920). Similarly, for Sample 2, the increases in response time were not significant across all conditions (congruent: *t*_(20)_ = −0.553, *p*_corrected_ = 0.707; control: *t*_(20)_ = 0.350, *p*_corrected_ = 0.627; incongruent: *t*_(20)_ = 0.210, *p*_corrected_ = 0.627).

The behavioral results indicated that drinking may affect the accuracy of subjects performing the Stroop color–word task. Compared to the pre-drinking phase, the accuracy decreased in the post-drinking phase. However, the impact varied between different Baijiu products. Specifically, in the incongruent condition, the effect of Product 1 was not statistically significant, while Product 2 caused a significant difference.

### 3.3. EEG Results

We chose the FCZ electrode to analyze, which is a representative channel in the Stroop task [38,44,47,48]. The FCZ channel is located at the mid-frontal cortex, a region that plays a crucial role in attention function [49].

**Figure 4:**
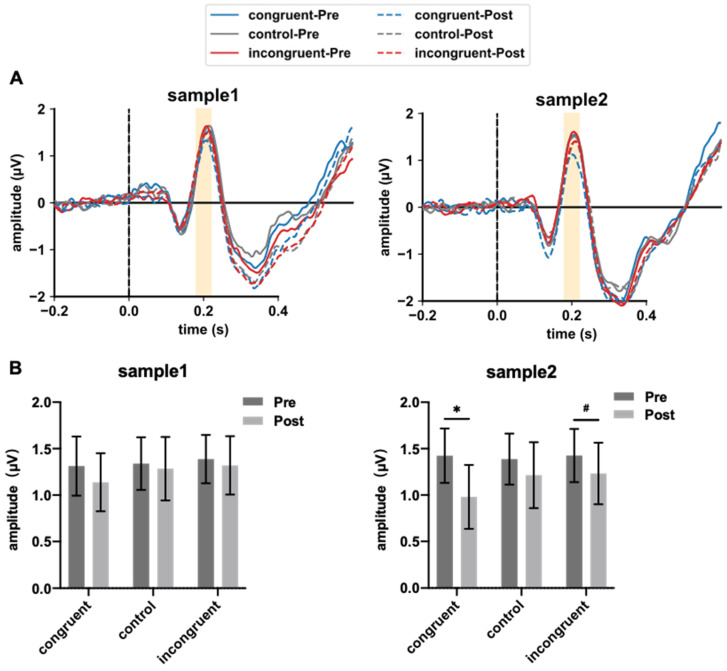
EEG results for the two samples under the three experimental conditions before and after drinking. (**A**) EEG waveforms of the FCZ electrode; (**B**) changes in P200 amplitude. One-sided paired *t*-tests with FDR correction for multiple comparisons, significance level set at 0.05, * indicates *p* < 0.05, # indicates *p* < 0.07.

Figure 4A depicts the EEG waves under three experimental conditions during the pre-drinking and post-drinking phases. The yellow-shaded areas indicate the P200 component, corresponding to the time window of 180–220 ms. It can be clearly seen that compared to the pre-drinking phase, there is a decreasing trend in EEG amplitude during the post-drinking phase across all three experimental conditions. Notably, this trend is observed for both samples within the same participant group, suggesting a degree of internal consistency in the effect.

Next, paired *t*-tests were performed on the amplitude of the P200 component before and after drinking under the three experimental conditions, and the FDR method was used for multiple corrections. The results are shown in Figure 4B. The reduction in the post-drinking stage for Sample 1 was not significant under all three conditions (congruent: *t*_(18)_ = 1.193, *p*_corrected_ = 0.358; control: *t*_(18)_ = 0.369, *p*_corrected_ = 0.358; incongruent: *t*_(18)_ = 0.625, *p*_corrected_ = 0.358). For Sample 2, the decrease was statistically significant under the congruent condition (*t*_(18)_ = 2.924, *p*_corrected_ = 0.014), not significant under the control condition (*t*_(18)_ = 1.178, *p*_corrected_ = 0.127), and marginally significant under the incongruent condition (*t*_(18)_ = 1.831, *p*_corrected_ = 0.063).

P200, which has been widely associated with attention processes, showed expected modulations under different aroma-type conditions. Alcohol consumption reduced the amplitude of the P200 component, although the degree of impact varied between different Baijiu samples. Under both congruent and incongruent conditions, the reduction in P200 amplitude for Sample 1 was not significant, whereas the reduction caused by Sample 2 was significant or marginally significant.

## 4. Discussion

To evaluate the post-drinking comfort quality of Baijiu quantificationally, this study utilized the Stroop color–word task to test the changes in consumer attention after drinking. It was found that Baijiu products with the same alcohol content but different aroma types had significantly distinct impacts on behavioral performance and neural responses. These findings suggest that EEG technology may serve as a useful tool for exploring the cognitive effects associated with different aroma types of Baijiu.

The two Baijiu samples used in this study had the same alcohol content but still produced different degrees of impact on brain attention function. We acknowledge that alcohol content is the key factor affecting attention, but other factors should not be overlooked, as evidenced in the behavioral and EEG results of the Stroop task in the current study. One possible explanation, which remains to be verified by future chemical analysis, is that differences in certain fermentation by-products (e.g., aldehydes or fusel oils), which are known to disrupt cellular function and exert acute neurotoxic effects [50,51], might contribute to the cognitive differences observed between samples.

Apart from alcohol content, the amount of alcohol consumed is also an important factor to consider. This study followed recommendations from the literature [39,40] to calculate the appropriate drinking amount for each subject based on their weight. However, the amount of alcohol consumed is also influenced by other factors such as genetics and body fat. For some individuals with higher alcohol tolerance, the administered dose may have produced only subtle cognitive changes, leading to non-significant effects in attention test after drinking. Moreover, the conclusions from the literature are based on Western subjects, and whether they can be generalized to Asian subjects remains a question. Therefore, future research can further explore individual drinking amounts of Chinese Baijiu products.

In the behavioral data analysis, we only found differences in accuracy scores between the two samples, with no significant differences in response time. One plausible explanation is that some participants may have prioritized accuracy over speed while performing the task, which could attenuate response-time effects; however, this possibility was not directly assessed and should be examined in future work.

A limitation of this study is the small number of Baijiu samples used for testing, although the two chosen samples were very similar in aroma type and had the same alcohol content. To further enhance the reliability and generalizability of the research conclusions, future studies could increase the variety of Baijiu samples or brands, such as samples with significantly different aroma types but the same alcohol content, or samples from the same aroma series with different alcohol contents but identical production processes.

The gender distribution in our sample (14 males, 7 females) was unbalanced, which may limit the generalizability of our findings. Previous studies have indicated potential gender differences in alcohol metabolism following alcohol consumption [52]. Although gender was not a primary factor considered in the current study, such imbalance might introduce unintentional biases regarding physiological and neurological responses. Therefore, future research employing larger sample sizes with balanced gender distribution is recommended to better assess potential sex differences and improve the robustness and generalizability of the conclusions drawn.

## Figures and Tables

**Table 1:** Accuracy and response time results of the two samples under three experimental conditions during the pre-drinking and post-drinking phases.

Sample Type	Behavior Indices	Pre-Drinking						Post-Drinking					
		Congruent		Control		Incongruent		Congruent		Control		Incongruent	
		*M*	*SD*	*M*	*SD*	*M*	*SD*	*M*	*SD*	*M*	*SD*	*M*	*SD*
sample 1	ACC	0.925	0.063	0.922	0.052	0.867	0.092	0.875	0.099	0.868	0.110	0.857	0.110
	RT (s)	0.519	0.080	0.543	0.089	0.552	0.090	0.524	0.100	0.533	0.088	0.552	0.104
sample 2	ACC	0.925	0.054	0.928	0.045	0.898	0.057	0.898	0.067	0.884	0.083	0.870	0.080
	RT (s)	0.504	0.083	0.522	0.086	0.531	0.085	0.511	0.095	0.518	0.101	0.528	0.097

## Data Availability

The original contributions presented in the study are included in the article, further inquiries can be directed to the corresponding authors.
